# ED50 of Intranasal Dexmedetomidine Sedation for Transthoracic Echocardiography in Children with or without a History of Cardiac Surgery for Cyanotic Congenital Heart Disease

**DOI:** 10.1155/2020/1349432

**Published:** 2020-12-12

**Authors:** HongBin Gu, YunAn Song, Jie Bai

**Affiliations:** ^1^Department of Anaesthesiology, Shanghai Children's Medical Centre, School of Medicine, Shanghai Jiao Tong University, Shanghai, China; ^2^Paediatric Clinical Pharmacology Laboratory, Shanghai Children's Medical Centre, School of Medicine, Shanghai Jiao Tong University, Shanghai, China

## Abstract

**Methods:**

This prospective single-blinded clinical trial included 72 ASA I-II stage children aged 1-36 months with cCHD who were scheduled to undergo TTE under sedation. Children were assigned to group A (*n* = 37) with a previous history of cardiac surgery and group B (*n* = 35) with no history of cardiac surgery. Doses of intranasal DEX were analyzed by up-down sequential allocation at an initial dose of 2.3 *μ*g/kg and an increase in steps of 0.2 *μ*g/kg. Intranasal DEXED50 values were analyzed by the up-and-down method of Dixon-Massey and probit regression to determine ED50 and 95% confidence interval (CI) for sedation. The time to effective sedation, time to regaining consciousness, vital signs, oxygen saturation, time of performing TTE, clinical adverse effects, and characteristics of regaining consciousness were compared between the two groups.

**Results:**

ED50 of intranasal DEX sedation was 2.530 *μ*g/kg (95% CI, 1.657-4.156) in group A and 2.500 *μ*g/kg (95% CI, 1.987-3.013) in group B. There was no significant difference in sedation onset time and time to regaining consciousness between the two groups. Additionally, no significant adverse hemodynamic or hypoxemic effect was observed. There was no significant difference in sedation-onset time and wake-up time between the two groups (15 ± 4 min *vs.*16 ± 5 min; 50 ± 11 min *vs.*48 ± 10 min). This trial is registered with the China Clinical Trials Registry (ChiCTR-IOR-1800015038).

**Conclusions:**

ED50 of intranasal DEX sedation for TTE is similar in children with and without a history of cardiac surgery for cCHD.

## 1. Introduction

Congenital heart disease (CHD) is the most common birth defect in China, with the prevalence of 26.6 per thousand, including 3.5 per thousand cases of major CHD [1]. Children with cyanotic CHD (cCHD) require repeated noninvasive examinations and even multiple surgeries during treatment, including cardiac catheterization, palliative surgery, and correction surgery, all of which have to be performed under anesthesia and sedation. Tetralogy of Fallot (TOF) is the most common form of congenital cyanotic heart disease and represents 7%-10% of all congenital heart defects. Within TOF, there is a broad spectrum of presentation: from the “PINK TET” to the cyanotic infant or the extreme variant of TOF with pulmonary atresia. The clinical manifestations of DORV are similar to TOF. In infants, repeated or long-term general anesthesia may affect their brain growth and development or result in the long-term outcome of attention deficit and hyperactivity disorder [2, 3]. Dexmedetomidine (DEX) is a common sedative drug used on the outpatient basis, knowing that it has a neuroprotective effect and little inhibition on respiration and circulation. However, there is no consensus about the optimal dose of DEX sedation in young children with an anesthesia history of correction surgery for cCHD. Some studies [4] reported differences in intranasal DEX dosage between patients with and without cCHD in nonsurgical settings, and others [5] reported the intranasal DEX dosage after surgery in patients with acyanotic CHD. Our previous study discovered that the dose of anesthetics or sedatives was not increased significantly in children who had a history of previous sedations [6]. The aim of the present study was to determine ED50 and ED95 of intranasal DEX sedation in children who had a history of cardiac surgery for cCHD, in an attempt to provide evidence-based clues for the rational clinical use of DEX for sedation in pediatric patients.

## 2. Materials and Methods

This prospective and single-blinded study was approved by the Institutional Review Board of Shanghai Children's Medical Center (SCMCIRB-K2017069) and registered with the China Clinical Trials Registry (ChiCTR-IOR-1800015038). Before the experiment, all instruments were adjusted and calibrated according to the standard requirements. A total of 72 children with cCHD who were scheduled to undergo daytime transthoracic echocardiography (TTE) were included in the study, and informed consent was obtained from their families or guardians, who were fully informed of the potential the adverse effects of sedatives and important matters that parents need to know during the monitoring process before initiation of the research. According to whether or not the patients had a history of cardiac surgery for CHD, they were divided into group A with a history of cardiac surgery (*n* = 37) and group B without a history of cardiac surgery (*n* = 35). The inclusion criteria were ASA I-II class pediatric patients of both genders aged 1-36 months with cCHD who were scheduled to undergo TTE. The exclusion criteria were children with abnormal liver and kidney function, a history of heart failure and/or preoperative bradycardia or arrhythmia who were administered with cardiotonic or antiarrhythmic drugs within one week, and had a history of allergy to the drug to be tested or recent upper respiratory tract infection.

### 2.1. Anesthesia and Management

All children were required to fast for 2 hours before the test. ASA was assessed by the anesthesiologist in a single-blind manner. Baseline vital signs including the heart rate (HR), noninvasive blood pressure (NBP), and transcutaneous oxygen saturation (SpO_2_) were measured and recorded. Based on the principles of upper and lower experiments and the results of previous preliminary experiments, the initial dose of DEX was set at 2.3 *μ*g/kg. The dose difference of each experimental subject was 0.2 *μ*g/kg. DEX (Jiangsu Hengrui Medicine Co., Ltd., Lianyungang, China) was administered intranasally. The DEX dose given to the first child was 2.3 *μ*g/kg. If this patient was evaluated as successful sedation, the drug dose of the next subject would be reduced by 0.2 *μ*g/kg, or increased by 0.2 *μ*g/kg if sedation failed. In case sedation failed, the anesthesiologist would give the subject 5 mg/kg phenobarbital sodium as a rescue sedation to ensure that the examination would be completed successfully. Each side of the nasal cavity was given 50% of the total dose of DEX. After drug administration, the nasal wing was pressed for several seconds to ensure that the drug was fully absorbed, and then the child was laid supine for 2 minutes. HR, BP, and SpO_2_ were monitored continuously during the examination. If the vital signs were lower or higher than 120% of the normal value, it was defined as abnormal. Five minutes after DEX administration, sedation depth was assessed using the Ramsay scale. If the Ramsay score was higher than 5 and the patient was able to receive an TTE examination without physical activity, sedation was considered successful; if the child woke up during TTE or was unable to complete the examination due to physical activity, sedation was considered a failure. The effective time of sedation (from drug treatment to the Ramsay score ≥ 5), the time to restore consciousness (from drug treatment to Aldrete improvement score ≥ 9), and related adverse reactions such as nausea, agitation, arrhythmia, delayed recovery of consciousness (more than 2 hours from TTE to recovery of consciousness), and respiratory depression were all recorded. After completing the ultrasound examination, the patient was retained in the post-anesthesia care unit and monitored continuously until the modified Aldrete score ≥ 9. After the situation was assessed by the anesthesiologist, the patient could leave the hospital.

### 2.2. Statistical Analysis

SPSS 17.0 software and Microsoft Excel were used for statistical analysis. The Kolmogorov-Smirnov test was used to test the normal distribution of quantitative data. Quantitative data are expressed as mean ± standard deviation (SD). Student *t*-test of was used for analysis and comparison of data that met the premise of analysis of variance. The Kruskal-Wallis test was used to compare nonnormally distributed data between the two groups. The qualitative data were analyzed by Pearson's chi-square test or Fisher's exact test. *P* < 0.05 was considered statistically significant.

The ED50 value and 95% CI of the experimental drug were calculated using the up-and-down sequence principle and the probit regression program [7, 8]. Based on the experiments performed this time, ED50 was calculated from the midpoint of all independent patient pairs involving a cross from failure to success. Finally, 37 patients in group A and 35 patients in group B were enrolled until ≥9 crossovers were received.

## 3. Results

Altogether, 72 children with cCHD (tetralogy of Fallot, double outlet right ventricle) were included in this research. Children with a history of correction or palliative surgery for CHD were included in group A, and those without such a history were included in group B. Of the 37 patients in group A, 12 had double outlet right ventricle (DORV) and 25 had tetralogy of Fallot (TOF), 2 patients were cyanotic due to incomplete corrective surgery; of the 35 patients in group B, all children had cyanotic heart disease, 7 had DORV and 28 had TOF. In group B, the left pulmonary artery diameters are from 0.5 cm to 1.2 cm, and right pulmonary artery from 0.6 cm to 1.2 cm. The diameter of the main pulmonary artery trunk is 1.0 cm-1.6 cm, and the size of the VSD defect is 0.85 cm-1.24 cm. The direction of VSD shunt in 22 children was bidirectional shunt, and the others were right-to-left shunt. The minimum pressure gradient between the right ventricle and the pulmonary artery was 20 mmHg, and the maximum was 44 mmHg. The minimum pulmonary artery velocity was 2.1 m/s, and the maximum was 3.15 m/s. No severe regurgitation was seen in the mitral and tricuspid valves of all children. Moreover, the minimum diameter of the pulmonary valve was 0.56 cm, and the maximum was 1.4 cm. The overriding DORV aorta was more than 50% in group B, of which 5 cases of VSD were subaortic, 1 case of VSD was distant from the both arteries, and one case was subpulmonic-VSD. No obvious coronary artery malformation was found in children.

The basic demographic characteristics of these children are shown in Table 1. Data are expressed as mean ± SD, and gender is expressed as frequency. There was no significant difference in the basic demographic data between the two groups.

Various hemodynamic parameters were continuously observed and recorded, and no significant difference was found between the observations at different time points in both groups. Except for SpO_2_, no significant difference was observed at the same time point between the two groups (Table 2). These results indicate that the hemodynamics of these children remained stable during the examination.

There was no significant difference in onset time of sedation, time of performing the TTE examination, and time of consciousness recovery between the two groups (all *P* > 0.05). The sedative effects of the whole study are shown in Table 3. The results showed that the onset time of sedation by intranasal DEX dripping was about 15 minutes; the time to complete TTE was about 17 minutes; and the time to fully regaining consciousness was about 50 minutes in both groups. As shown in Table 3, the children regained consciousness within 30 minutes after completion of the examination. All patients with sedation failure were administered with an intramuscular injection of sodium phenobarbital (5 mg/kg) as a remedial measure.

The adverse effects observed in the experiment are recorded. No serious adverse events such as respiratory depression, cardiovascular depression, and hypoxia were observed throughout the study, and no significant difference in the incidence of adverse reactions was observed between the two groups. However, mild adverse reactions including reduced HR occurred in 3 patients in group A and 4 patients in group B. The overall incidence of bradycardia in all patients was 9.7%.

As these adverse reactions did not cause obvious harms to the patients, no special treatment was prescribed until they returned to normal before leaving the hospital. Nevertheless, special attention was still required to the meticulous monitoring of the children throughout the TTE process.

**Figure 1:**
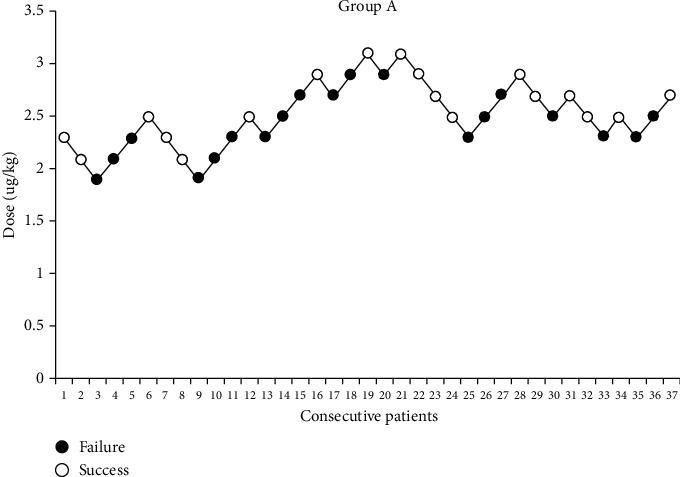
Up-and-down sequential allocation analysis of intranasal dexmedetomidine in the two groups.

**Figure 2:**
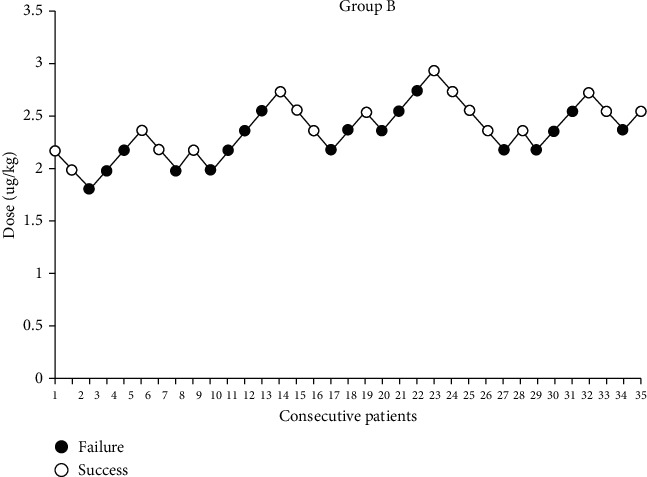
Up-and-down sequential allocation analysis of intranasal DEX was performed in all pediatric patients receiving sedation for TTE in both groups. The dose increment/decrement was 0.2 *μ*g/kg. The calculated ED50 was 2.486 *μ*g/kg (2.530 *μ*g/kg) in group A and 2.485 *μ*g/kg (2.500 *μ*g/kg) in group B.

Within group A and group B, 19 and 18 patients, respectively, needed to use phenobarbitone due to sedation failure. Dixon and Massey's method showed that ED50 in group A was 2.486 *μ*g/kg (2.530 *μ*g/kg) and 2.485 *μ* g/kg (2.500 *μ*g/kg) in group B. Probit regression analysis showed that ED50 and ED95 in group A were almost the same as those in group B [2.530 (1.657-4.156) and 3.627 (3.039-12.424))*μ*g/kg *vs.* 2.500 (1.987-3.013) and 3.358 (2.920-11.106) *μ*g/kg, *P* > 0.05]. Moreover, the upper limit dose of ED95 exceeded the safe dose range. The actual reaction curve and drug dosage are shown in Figures 1 and 2.

## 4. Discussion

Chloral hydrate remains one of the common sedatives for various examinations in pediatric outpatients. However, these sedative drugs may cause ineffective sedation, respiratory depression, or nausea and vomiting [9, 10]. In addition, they also have potential neurotoxic hazards. DEX is known to have a neuroprotective effect and can be more easily absorbed when administered intranasally because of the rich capillaries in the nasal cavities, offering a bioavailability of 65% and good compliance in young children [11, 12]. Unlike chloral hydrate, dexmedetomidine is a new type of sedative suitable medicine for outpatients, which has little respiratory and circulatory system suppression [13, 14]. There is a high incidence of perioperative adverse events affecting the upper respiratory tract in children with CHD, especially cCHD. Zhang et al. [15] found that intranasal DEX dripping could not only reduce the incidence of perioperative adverse events of the upper respiratory tract and improve oxygenation in CHD children but reduce adverse effects arising from various stimuli. Based on the bioavailability of DEX and our initial pilot results, we started with the dose of intranasal DEX at 2.3 *μ*g/kg [16, 17] .

To improve the research efficiency and simplify the research process, we used the sequential method in this study, knowing that it can save the sample size, although a small sample size may cause some limitations [18, 19]. According to the principle of the Dixon-Keilin method, there should be more than six crossover points. Actually, the Uniform Design Method (UDM) as a sequential method has an intrinsic limitation; i.e., the drug dosage of the previous patient decides that of the subsequent patient. To guarantee the accuracy and precision of the experiment, we expanded our sample size by selecting more than 10 crossover points. Finally, we included 37 patients in group A and 35 patients in group B. Therefore, our results are more evidence-based and more statistically significant. It is worth noticing that ED50 is only a pharmacological concept which refers to the drug concentration causing 50% inhibition of the desired activity. It is a result of probability theory with instructive significance but does not reflect the true dosage of the drug. According to the sequential formula of calculating ED50 in the Dixon-Massey method and probit regression, the ED50 result was about 2.5 *μ*g/kg in both groups. It can not only reduce the fear and pain of the children but avoid the first pass effect of oral medication in the liver and intestine.

Cyanotic CHD is a special type of heart disease containing a variety of disease spectra, each with different circulatory physiological characteristics. Some often require multiple operations such as cardiac catheterization and long-term follow-up after surgery [20, 21]. In the subconscious mind of many anesthesiologists, children who have experienced multiple episodes of anesthesia or sedation will develop resistance to narcotics or sedatives and need to give larger drug doses to achieve a suitable sedative or anesthetic state. Our previous research found that children who had experienced multiple episodes of sedation did not require a significantly larger dose of sedatives. To the best of our knowledge, few studies have reported effective or optimal doses of sedatives in cCHD patients with a previous history of surgery. The present study found no significant difference in the effective dose between the two groups, suggesting that the pathophysiological state of patients with cCHD has a greater impact on the drug efficacy than surgical and perioperative factors.

It is interesting to find that the ED50 value is quite similar or almost the same between children with cCHD who had a past history of surgery and those who had no past history of surgery. This result is clearly different from that reported by Gu et al. [6], who found that ED50 in CHD children after cardiac surgery was two-fold higher than that in normal children, while Liu et al. [5] reported that ED50 in children with cCHD was higher than that in children with non-cCHD (3.2 *μ*g/kg *vs.* 1.9 *μ*g/kg). The discrepancies between our study and their studies may be attributed to the following reasons: (1) Yang et al. did not clearly define the types of cCHD in their study, while we only studied TOF and DORV, and therefore the disease is more uniform and the disease status is more consistent in our study. (2) The children in our study were observed in a specially designed room with a quiet indoor environment and soft lighting after drug administration, which is easier for children to fall asleep. (3) The steps of the experimental drugs were different (0.2 *μ*g/kg in our experiment, 0.5 *μ*g/kg in Liu's, and 0.25 *μ*g/kg in Yang's). (4) Lastly, the ultrasound performer is more stable and more consistent with the degree of sedation.

An overview of the literature shows that surgery has little impact on ED50 of cCHD, which may suggest a meaningful potential implication: the intrinsic internal milieu of cCHD children makes their requirement for drug dosage different from children with other diseases, whether or not there is a history of surgery and sedation. The possible reasons are as follows: first, anatomically, there is a right-to-left shunting or obstruction in cCHD, which then produces a unique change in the internal milieu, affecting the metabolism and pharmacokinetics [22], or even the growth and development of the central nervous system (CNS) [23], which further affects the depth and duration of sedation, and finally leading to the requirement of the patient for different anesthetics and sedatives.

In the present study, the mean spontaneous eye-opening and waking-up time was 50 minutes in both groups, without using any stimulant to speed up recovery. The adverse reaction rate was about 10%, with no significant hemodynamic change and respiratory depression observed. No airway, oxygenation, or hemodynamic intervention was adopted in any of the patients in both groups, except for continuous monitoring to prevent any unexpected accident from occurring. There was a mild-to-moderate decrease in HR, and only about 5% of the patients had an HR < 80 bpm (bradycardia) [24]. After completion of TTE under intranasal DEX sedation, the depth of sleep was in an arousable state and consciousness recovered stably in all patients. This simple and safe sedation method is acceptable by both the doctor and the patients' families.

There are some limitations in this study. First, we did not make a deep analysis on the interval between the previous surgery and the present procedure. Knowing that it may be an important influencing factor, we will make more detailed stratification in our ongoing work. In addition, it is a pity that we failed to make comparisons with children without cCHD after surgery under the same experimental conditions. Finally, the anesthetic drugs used during surgery were not accurately stratified and the method of cardiopulmonary bypass was not taken into consideration, knowing that they may also important factors that may affect the results.

## 5. Conclusions

In summary, this is the first study reporting the postoperative ED50 in young children who underwent TTE under DEX sedation after cardiac surgery for cCHD. Intranasal administration of dexmedetomidine is safe for transthoracic ultrasonography in children with cyanotic heart disease. Our results demonstrated that the presence or absence of a past history of cardiac surgery had little impact on the dose of intranasal DEX sedation for TTE in children with cCHD. Therefore, clinicians should be prudent to increase the dose of sedatives for anesthesia or sedation in such children. In addition, they should not only pay attention to hemodynamic changes arising from the use of anesthetics or sedatives but watch for the possible pathological effect of the disease itself on the drug dosage.

## Figures and Tables

**Table 1:** Demographic characteristics of children at baseline.

Group	Gender (M/F)	Age (months)	Height (cm)	Weight (kg)	Body surface area (m^2^)
Group A	20/17	17.2 ± 4.24	109.4 ± 6.1	16.1 ± 5.7	0.55 ± 0.11
Group B	19/16	18.3 ± 3.47	110.1 ± 9.8	16.4 ± 5.3	0.56 ± 0.14

Note: data are presented as mean ± SD or number (*n*/*n*). Group A with a heart surgery history and group B without a heart correction surgery. There was no significant difference between the two groups.

**Table 2:** Demographic data and baseline scores after intranasal dexmedetomidine sedation.

	Before sedation (baseline value)		During sedation		During regaining consciousness	
	Group A	Group B	Group A	Group B	Group A	Group B
HR (/min)	112 ± 16	106 ± 15	108 ± 16	104 ± 14	118 ± 14	115 ± 17
SpO_2_	91.6 ± 3.2	82.5 ± 4.2^#^	94.2 ± 2.1	87.2 ± 3.1^#^	91.2 ± 3.1	88.3 ± 2.7^#^
SBP (mmHg)	75.3 ± 16.4	77 ± 18.3	79 ± 15.2	85 ± 16.7	79 ± 14.1	87 ± 18.1
DBP (mmHg)	46.2 ± 12.2	51 ± 13.4	50 ± 12.2	54 ± 13.5	50 ± 11.3	56 ± 15.5

Note: data are presented as mean ± SD. SBP: systolic blood pressure; DBP: diastolic blood pressure. HR: heart rate; SpO_2_: pulse oxygen saturation. ^#^Intergroup difference, *P* < 0.05. There was a significant difference in SpO_2_ between the two groups because these children were divided according to the surgery: group A with a history of heart surgery and group B without a history of heart surgery.

**Table 3:** Time of achieving effective sedation, performing TTE, and regaining consciousness.

	Time of achieving effective sedation (min)	Time of performing TTE (min)	Time of regaining consciousness (min)	Acceptance of guardian (*n*)	Acceptance of clinician (*n*)
Group A	15 ± 4	4.5 ± 2	50 ± 11	37	37
Group B	16 ± 5	4.5 ± 1.5	48 ± 10	35	35

TTE: transthoracic echocardiography.

## Data Availability

The datasets used and analyzed during the current study are available from the corresponding author upon request.
